# Paternal obesity induces metabolic and sperm disturbances in male offspring that are exacerbated by their exposure to an “obesogenic” diet

**DOI:** 10.14814/phy2.12336

**Published:** 2015-03-24

**Authors:** Tod Fullston, Nicole O McPherson, Julie A Owens, Wan Xian Kang, Lauren Y Sandeman, Michlle Lane

**Affiliations:** 1 Discipline of Obstetrics & Gynaecology, Robinson Research Institute, Research Centre for Reproductive Health, School of Paediatrics and Reproductive Health, University of AdelaideAdelaide, SA, Australia; 2 Freemasons Foundation Centre for Men's Health, The University of AdelaideAdelaide, SA, Australia; 3 Monash IVF GroupMelbourne, Vic., Australia

**Keywords:** Metabolic syndrome, obesogenic environment, paternal obesity, paternal programming, second-hit, subfertility

## Abstract

Obesity and related comorbidities are becoming increasingly prevalent globally. In mice preconception paternal exposure to a high fat diet (HFD) impairs the metabolic and reproductive health of male offspring, despite their control diet (CD) consumption. However, offspring share lifestyle, including diet, with parents. We assessed if male offspring from HFD fathers have a heightened susceptibility to HFD-induced metabolic and reproductive derangements. This 2 × 2 design saw founder males (F0) and their offspring (F1) fed either a HFD or a nutritionally matched CD. Regardless of paternal diet, HFD fed male offspring had greater total body weight and adiposity. Offspring sired by a HFD male and fed a HFD were the heaviest, had the greatest adiposity and had the greatest concentration of serum cholesterol, triglyceride, HDL, and NEFA compared with CD sired/fed littermates. A synergistic increase in serum insulin was unmasked by both father/son HFD consumption, concomitant with increased sera glucose. Either a paternal or offspring HFD was associated with similar reductions to offspring sperm motility. Whereas sperm ROS concentrations and sperm–oocyte binding saw detrimental effects of both F0 HFD and F1 HFD with an interaction evident between both, culminating in the most impaired sperm parameters in this group. This indicates that metabolic and fertility disturbances in male offspring sired by HFD fathers are exacerbated by a “second-hit” of exposure to the same obesogenic environment postnatally. If translatable to human health, this suggests that adverse reproductive and metabolic outcomes may be amplified across generations through a shared calorie dense diet, relevant to the current worldwide obesity epidemic.

## Introduction

Obesity and its associated health comorbidities (*e.g.,* insulin resistance and type 2 diabetes) are currently at unprecedented rates of prevalence worldwide (Ng et al. 2014). More than 60% of all adults are classified as overweight or obese in most westernized societies, and as the prevalence of obesity increases it is responsible for an ever larger proportion of the overall burden of disease (Nguyen and El-Serag 2010; Ng et al. 2014).

It has been well documented that maternal obesity predisposes offspring to obesity, independently of genetic inheritance (Fall 2011), thus providing the potential for intergenerational amplification of obesity and its comorbidities (Dunn and Bale 2009, 2011). Rodent models of diet-induced obesity with or without altered glucose homeostasis in the father have also been shown to impair the metabolic and reproductive health of both male and female offspring via nongenetic pathways (Ng et al. 2010; Fullston et al. 2012, 2013). This provides evidence for paternal initiated transgenerational transmission of obesity (Fullston et al. 2013), altered glucose homeostasis (Ng et al. 2010; Fullston et al. 2013) and reproductive dysfunction (Fullston et al. 2012) for up to two generations through both maternal and paternal F1 lineages in rodents (Fullston et al. 2012, 2013). Furthermore, a paternal low protein diet has been demonstrated to cause hypotension, elevated heart rate, vascular dysfunction, impaired glucose tolerance, and increased adiposity in male offspring (Watkins and Sinclair 2014).

Human epidemiological investigations provide evidence for male transmission of noncommunicable diseases to descendants. For example, in a study of three generations within a population from Northern Sweden (Överkalix), a surfeit of grandpaternal food is associated with reduced survivability (Bygren et al. 2001) and an increased risk of diabetes (Kaati et al. 2002) in their grandchildren. Early life onset of grandpaternal smoking is also associated with increased grandson BMI (Pembrey et al. 2006). Furthermore, grandmaternal exposure during the Dutch famine lead to increased body weight and incidence of obesity in grandchildren, when transmission occurred via the sons who were *in utero* during the famine, but, interestingly, not via daughters (Veenendaal et al. 2013). In addition, children born to men of advanced paternal age have an increased risk of schizophrenia and autism, potentially via an increased sperm borne mutation load (Kong et al. 2012).

Although a father's BMI is associated with increased BMI in his children (Danielzik et al. 2002; Li et al. 2009; Whitaker et al. 2010; Freeman et al. 2012) this phenomenon is usually attributed to a shared genetic predisposition and/or an “obesogenic” environment shared by the father and his children. Indeed dietary habits can be intergenerational (Wahlqvist et al. 2014). The hypothesis of the thrifty phenotype was proposed to explain the phenomena whereby fetal exposure to undernourishment *in utero* (*i.e.,* by growth restriction or placental insufficiency) developmentally programs a thrifty phenotype in the fetus. But, if subsequently such offspring are born into a nutrient normal or nutrient rich environment rather than a deprived one, their risk of metabolic syndrome is greatly increased (Hales and Barker 1992). Another refinement of this hypothesis posits that the thrifty phenotype occurs as a predictive adaptive response, whereby an unborn offspring is programmed to “anticipate” the parental environment, making it better adapted to that environment as an adult (Gluckman et al. 2005). In this study, offspring might be expected to undergo a predictive adaptive response and thus be better adapted to the calorie dense diet, although little is known about whether this adaptive phenomenon can occur via paternal transmission.

Given that a paternal high fat diet in rodents predisposes male offspring to metabolic (Fullston et al. 2013) and reproductive pathologies (Fullston et al. 2012; McPherson et al. 2014) when fed a standard diet, it remained to be tested what effect the consumption of the same diet would have on metabolic and reproductive pathologies of offspring. Thus, we investigated to what extent the consumption of the same obesogenic diet as their father might exacerbate or protect these male offspring from their predisposed metabolic and reproductive phenotypes.

## Materials and Methods

### Animal model and feeding regimens

Using our established model of paternal HFD-induced obesity in the absence of impaired glucose homeostasis (Bakos et al. 2011b; Mitchell et al. 2011; Palmer et al. 2011; Fullston et al. 2012, 2013), founder (F0) C57Bl6 male mice were given *ad libitum* access to water and were randomly allocated to either a control diet (CD; *n *=* *6; 6% fat, SF00-219, Specialty Feeds, Glen Forrest, Australia; Table1) or a high fat diet (HFD, *n *=* *8; 21% butter fat, SF00-219, Specialty Feeds, Glen Forrest, Australia; Table1) from 5 weeks until 17 weeks of age, with body weight measured weekly. CD/HFD F0 males were mated in two consecutive rounds with 8-week-old normal weight naturally cycling females to produce F1 offspring in the last week of the diet regimen. The F0 males were humanely killed at 17 weeks of age (12 weeks of diet) and body composition was measured post mortem by dissection. Peri-renal, retro-peritoneal, omental, dorsal, and gonadal fat pads were dissected/weighed, as well as vastus lateralis muscle, soleus muscle, liver, pancreas, and kidneys.

**Table 1:** Composition of control diet (CD) and high fat diet (HFD)

Ingredient (%)	CD (SF04-057)	HFD (SF00-219)
Sucrose	34.1	34.1
Casein acid	19.5	19.5
Canola oil	6.0	
Clarified butter fat		21
Cellulose	5.0	5.0
Wheat starch	30.5	15.5
Vitamins/Minerals	4.9	4.9
Digestible energy (kJ g^-1^)	16.1	19.4
Digestible energy from lipids (%)	21.0	40.0
Digestible energy from protein (%)	14.0	17.0

Mean (±SEM) *ad libitum* consumption of feed per 24 h was 3.44 ± 0.05 g (*n *=* *6 male mice measured for daily feed intake over a 10 week period).

The HFD diet is a Harlan Teklad TD 88137 equivalent and is intended to mimic a western style fast food diet.

Founder females were monitored daily from day 18 of pregnancy and litter sizes were equalized to *n *=* *8 between treatment groups by either sacrifice for litters *n *>* *8, whereas litters with less than eight pups were excluded from analysis. This was to minimize between litter variation in regards to competition for nutrients *post partum*.

F1 offspring were allocated in a 2 × 2 feed treatment, whereby 2 F1 littermates sired by either CD or HFD fed F0 mice (F0 *n *=* *9 represented per F1 diet; *i.e., n *=* *4 CD F0, *n *=* *5 HFD F0) were themselves allocated to either the same CD (*n *=* *34 males) or HFD (*n *=* *33 males; 1 male from a sibling pair died upon diet allocation). F1 mice were fed *ad libitum* with free access to water from 5 weeks of age for 16 weeks. Animals are referred to throughout as:

CD/CD – CD fed F0 then CD fed F1
CD/HFD – CD fed F0 then HFD fed F1
HFD/CD – HFD fed F0 then CD fed F1
HFD/HFD – HFD fed F0 then HFD fed F1

F1 total body weight was measured weekly and body composition determined by postmortem dissection at 21 weeks of age (16 weeks post diet challenge). Tissues and organs of all animals were dissected and weighed postmortem.

Mice were obtained and housed by the University of Adelaide (Laboratory Animal Services, Adelaide, Australia) being maintained at 24°C on a 14 h light, 10 h dark illumination cycle. The animal ethics committee of the University of Adelaide approved all experiments, and the animals were handled in accordance with the Australian Code of Practices for the Care and Use of Animals for Scientific Purposes.

### Blood serum metabolite and hormone analysis

Animals were fasted overnight and whole blood samples were taken via cardiac puncture under isoflurane anesthetic and serum was isolated by centrifugation. Serum glucose, cholesterol, nonesterified-free fatty acids (NEFA), triglycerides, and high-density lipoproteins (HDL) were measured using enzymatic analysis (De Blasio et al. 2007) and a Cobas Mira automated system (Roche Diagnostics, Basel, Switzerland).

Enzyme-linked immunosorbent assays measures blood serum insulin (Ultra-sensitive mouse insulin ELISA, Crystal Chem, Downers Grove, IL) and leptin (Mouse leptin ELISA, Crystal Chem).

### Metabolic assessment

Littermates were fed either a CD or HFD (*n *=* *14–20 male offspring/diet/F_0_ diet; with *n *=* *4 CD F0 and *n *=* *5 HFD F0 founders represented) and all F1 animals were subjected to all metabolic tests. Glucose tolerance tests (GTTs) were performed at 12 and 18 weeks of age (7 and 13 weeks diet treatment, respectively) after 6 h of fasting, by intraperitoneal (ip) injection of 2 g kg^−1^ of 25% glucose solution (Sigma, St Louis, Missouri) and insulin tolerance tests (ITTs) were conducted at the same ages, under fed conditions, by ip injection of 0.75 IU kg^−1^ insulin (Actapid, Novo Nordisk, Bagsvaerd, Denmark). Tail blood glucose concentrations were measured using a glucometer (Hemocue, Angelholm, Sweeden) at timepoints 0 (prebolus basal), 15, 30, 60, and 120 min. Data are expressed as 1) mean blood glucose concentration per group using area under curve (AUC; glucose challenge) or area above the curve (AAC) analysis (insulin challenge) or 2) time of peak glucose response for GTT and minimum blood glucose concentration recorded during an ITT. Note that both peak glucose (GTT) and minimum glucose concentration (ITT) were limited to the standard timepoints used in these assays.

### Male reproductive measures

#### Testosterone concentration

Blood serum testosterone concentration was determined using a competitive enzyme immunoassay (R&D Systems, Minneapolis, Minnesota).

#### Sperm count, motility, and vitality

Spermatozoa collected from the vas deferens and cauda epididymis were extracted into GIVF medium (Vitrolife AB, Gothenburg, Sweden) and allowed to swim out for 10 min at 37°C in 5% O_2_, 6% CO_2_, and 89% N_2_. Sperm concentration was determined using a Neubauer hemocytometer as recommended by the World Health Organization laboratory manual for the examination of human semen and sperm–cervical mucus interaction (WHO and World Health Organisation 2010). Using 40× magnification at 20–25°C (*i.e.,* room temperature), sperm motility was determined manually from duplicate measures of 200 sperm (Bakos et al. 2011b) and expressed as a percent of motile sperm for each sample. Vitality was assessed on >100 sperm per sample by the proportion of sperm that were propidium iodide (PI) positive as a total of sperm counted after a 5 min incubation with PI.

#### Sperm intracellular reactive oxygen species

As a broad measure of multiple intracellular reactive oxygen species (ROS) concentrations were assessed in progressively motile spermatozoa. Analysis was conducted on live motile sperm because the dye (2′,7′-dichlorodihydrofluorescein diacetate; DCFDA; Sigma) requires intracellular esterase cleavage to enable detection of ROS. The dye fluoresces when bound to intracellular ROS as previously described (Nasr-Esfahani et al. 1990; Bakos et al. 2011b; Fullston et al. 2012; Kazama and Hino 2012; Fatemi et al. 2013). Briefly, sperm were incubated with 5 *μ*mol L^−1^ DCFDA for 15 min at 37°C, washed twice and examined using a photometer attachment on a fluorescent microscope to derive a fluorescence reading for individually imaged sperm. The DCFDA fluorescence used to measure ROS concentration for each spermatozoa was expressed as relative to CD/CD. A minimum of 30 motile sperm were measured per diet group (Mean 41.5 ± 6.6), as described previously (Bakos et al. 2011b; Fullston et al. 2012).

#### Sperm binding

Zona pellucida binding by sperm was measured using cumulus-enclosed ovulated oocytes, collected from 4-week-old C57Bl6 × CBA females 12 h post superovulation induced with an ip injection of pregnant mare's serum gonadotropin (PMSG; Folligon; Intervet, Bendigo, Australia) and human chorionic gonadotrophin (hCG; Pregnyl; Organon, Oss, The Netherlands) delivered 48 h apart (Gardner et al. 2004). Vas deferens sperm samples were capacitated for 1 h in GIVF medium (Vitrolife) and 1 × 10^6^ motile sperm per mL, coincubated with COCs (*n *≥* *30 oocytes per male; Mean 34.3 ± 4.0) in GIVF at 6% CO_2_, 5% O_2_, and 89% N_2_ at 37°C. for 4 h. Oocytes were then incubated for 3 min in bisbenzamide (25 *μ*g mL^−1^), followed by imaging under UV light and the number of sperm bound to the zona pellucida measured by counting sperm nuclei and expressed as a proportion of F1 mice sired by CD F0 and fed a CD.

### Statistics

F0 outcomes were analyzed using a one way ANOVA, or a Mann Whitney U test for nonparametric data. Differences between groups for binomial data were assessed by Fisher's exact test. Analysis of measures in offspring was performed by linear mixed models (LMM; 2-way analysis of variance; ANNOVA) or for repeated measures (i.e., ITT/GTT) a mixed models repeated measures 2-way ANNOVA. In the 2 × 2 dietary model father's diet and offspring diet as fixed factors and father ID as a random factor. This analysis allowed for the comparison of main effects for founder diet and offspring diet on all measures and whether significant interactions occurred, while controlling for offspring variation from the same Founder. Between treatment differences were assessed using a least significant difference post hoc analysis. Levels of significance were *P *≤* *0.05.

## Results

### A HFD-induced obesity without impaired glucose homeostasis (at 12 weeks of age) in Founder males (F0)

After 6 weeks of diet treatment, HFD F0 males had increased body weight (vs. CD; *P *<* *0.050), which persisted through the mating period (13–17 weeks of age) until the end of the 12-week-diet regimen (17 weeks old), compared with CD males (32.7 ± 0.73 g vs. 28.18 ± 0.87 g, respectively; *P *<* *0.050). GTT (assessed by AUC of blood glucose concentration) 7 weeks post diet intervention (12 weeks of age) was not different between CD and HFD F0 males (CD: 744 ± 91 mmol L^−1^.min; HFD: 850 ± 113 mmol L^−1^ min; *P *=* *0.482). After 12 weeks of diet exposure (17 weeks of age), adiposity was increased in HFD F0 males compared with CD (+77.3% in absolute mass of adipose, *P *<* *0.001; and +53.0% as a proportion of total body weight, *P *<* *0.001). HFD F0 males fasting serum lipids were elevated compared with CD F0 males (*cholesterol* CD: 3.52 ± 0.13 mmol L^−1^ vs. HFD: 5.40 ± 0.31 mmol L^−1^, *P* < 0.001; *high-density lipoproteins:* CD 3.08 ± 0.09 mmol L^−1^ vs. HFD: 4.59 ± 0.23 mmol L^−1^, *P* < 0.001; *free fatty acids:* CD 1.05 ± 0.06 *μ*Eq mL^−1^ vs. HFD: 1.51 ± 0.10 mEq L^−1^, *P* < 0.01; *Triglycerides:* CD 0.56 ± 0.06 mmol L^−1^ vs. HFD: 1.04 ± 0.11 mmol L^−1^, *P* < 0.010). Whereas fasted blood glucose concentration was not different (CD 9.19 ± 0.49 mmol L^−1^ vs. HFD 10.13 ± 0.49 mmol L^−1^, *P* = 0.183).

Consistent with our previous studies (Bakos et al. 2011b; Palmer et al. 2011; Fullston et al. 2012, 2013), HFD F0 males had reduced sperm motility (CD: 84.2 ± 2.1% vs. HFD: 72.3 ± 2.1%, *P *<* *0.010) and increased intracellular sperm DCFDA fluorescence as a measure of ROS concentrations (CD: – 176 ± 3 relative fluorescence units (rfu) vs HFD: – 242 ± 5 rfu, *P *<* *0.001). Sperm count was not effected (CD: 1.66 ± 0.12 × 10^6^ sperm mL^−1^ vs. HFD: 1.64 ± 0.07 × 10^6^ sperm mL^−1^, *P *=* *0.881) nor was serum testosterone concentration (CD: 2.95 ± 0.86 ng mL^−1^ vs. HFD: 4.66 ± 1.10 ng mL^−1^, *P *=* *0.324), compared with CD F0 males.

### HFD F0 sired and HFD fed F1 male offspring have the greatest adiposity

**Figure 1:**
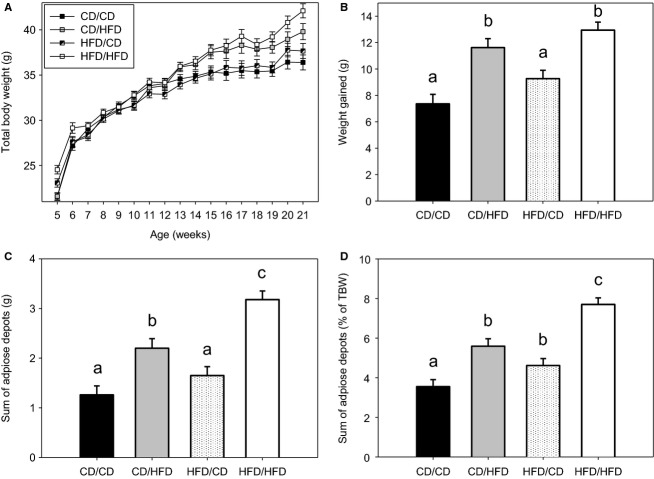
Total body weight and adiposity of F1 male offspring fed a CD/HFD fathered by CD/HFD F0 males. (A) Total body weight during the dietary challenge, (B) total weight gained during the dietary challenge, sum of adipose depots dissected postmortem upon completion of the dietary challenge expressed in both (C) absolute terms and (D) as a proportion of total body weight are depicted for F1 males for the groups CD/CD (CD fed F1 sired by a CD fed F0; *n *=* *14), CD/HFD (HFD fed F1 sired by a CD fed F0; *n *=* *14), HFD/CD (CD fed F1 sired by a HFD fed F0; *n *=* *20) and HFD/HFD (HFD fed F1 sired by a HFD fed F0; *n *=* *19). Data are presented as means ± SEM and different letters represent statistically distinct groups (*P *<* *0.05).

F0 diet did not affect total body weight or total weight gain of F1 male offspring at or by 21 weeks of age (*P* > 0.05, Fig.1A; Table2). Whereas a HFD in F1 male offspring, regardless of F0 diet, increased both total body weight at 21 weeks of age and weight gain from 5 to 21 weeks of age compared with sibling males fed a CD (*P* < 0.05, Fig.1A and B; Table2). Of note, the HFD/HFD F1 males had the greatest total body weight at 21 weeks of age and gained the most weight gain from 5 to 21 weeks of age (Fig.1; Table2).

**Table 2:** Summary of F_1_ male body composition, sera metabolites, and glucose homeostasis measures at age 21 weeks of age, 15 weeks post diet challenge

F0 male diet	CD	CD	HFD	HFD
F1 male diet	CD	HFD	CD	HFD
F1 Male offspring	*n *=* *14	*n *=* *14	*n *=* *20	*n *=* *19
Total body weight (g)^1^	36.4 ± 0.8 a	39.8 ± 0.9 bc	37.7 ± 0.8 ab	42.1 ± 0.8 c
Adiposity				
Peri-renal (g)	0.05 ± 0.01 a	0.09 ± 0.01 a	0.09 ± 0.01 a	0.14 ± 0.01 b
Retro peritoneal (g)	0.13 ± 0.04 a	0.23 ± 0.04 a	0.15 ± 0.04 a	0.34 ± 0.04 b
Omental (g)	0.36 ± 0.07 a	0.54 ± 0.07 ab	0.45 ± 0.07 a	0.73 ± 0.07 b
Dorsal (g)	0.20 ± 0.02 a	0.29 ± 0.03 bc	0.26 ± 0.02 ab	0.36 ± 0.02 c
Gonadal (g)	0.52 ± 0.10 a	1.06 ± 0.10 b	0.73 ± 0.10 a	1.60 ± 0.09 c
Organ weights				
Vastus Lateralis (mg)	195 ± 9.0	217 ± 9.6	208 ± 9.2	204 ± 8.7
Soleus (mg)	8.4 ± 0.9	10.6 ± 1.0	10.3 ± 0.9	10.3 ± 0.9
Liver (g)	1.34 ± 0.09 a	1.53 ± 0.09 ab	1.29 ± 0.09 a	1.63 ± 0.09 b^2^
Pancreas (g)	0.14 ± 0.01	0.17 ± 0.01	0.16 ± 0.01	0.17 ± 0.01
Kidneys (g; L + R)	0.51 ± 0.01	0.53 ± 0.01	0.48 ± 0.01	0.50 ± 0.01
Blood metabolites				
Glucose (mmol L^−1^)	6.41 ± 1.19 a	7.58 ± 1.13 a	9.70 ± 0.95 ab	11.48 ± 0.83 b
Cholesterol (mmol L^−1^)	3.68 ± 0.46 a	5.33 ± 0.44 bc	4.73 ± 0.37 ab	6.43 ± 0.32 c
Triglyceride (mmol L^−1^)	0.67 ± 0.16 a	1.25 ± 0.15 bc	0.92 ± 0.13 ab	1.58 ± 0.11 c
HDL (mmol L^−1^)	3.28 ± 0.37 a	4.85 ± 0.35 bc	3.88 ± 0.30 ab	5.17 ± 0.30 c
NEFA (mEq L^−1^)	1.32 ± 0.26 a	1.88 ± 0.25 a	1.73 ± 0.21 a	2.35 ± 0.19 b
Leptin (ng mL^−1^)	4.95 ± 1.05 a	4.54 ± 1.05 a	9.69 ± 0.86 b	8.56 ± 0.95 b
Insulin (ng mL^−1^)	0.12 ± 0.02 a	0.13 ± 0.02 a	0.14 ± 0.02 a	0.20 ± 0.02 b
Glucose Homeostasis				
GTT AUC (mmol L^−1^ min) – 12 weeks	625.1 ± 107.7	725.3 ± 104.7	508.0 ± 89.7	629.4 ± 89.7
GTT AUC (mmol L^−1^ min) – 18 weeks	548.4 ± 95.9	644.2 ± 98.7	696.8 ± 81.1	710.2 ± 82.2
ITT AAC (mmol L^−1^ min) – 12 weeks	259.1 ± 41.5 ab	191.5 ± 46.8 ab	314.3 ± 32.4 a	165.9 ± 33.2 b
ITT AAC (mmol L^−1^ min) – 18 weeks	202.0 ± 50.6 ab	164.0 ± 50.6 ab	273.0 ± 42.1 a	130.1 ± 44.6 b

CD, control diet; HFD, high fat diet.

1 Total body weight (TBW) was measured prior to the overnight fast that preceded post mortem examination. Different letters represent statistically distinct groups (*P *<* *0.05). HDL high-density lipoprotein. NEFA nonesterified fatty acid. GTT glucose tolerance test. AUC area under the curve analysis. ITT insulin tolerance test. AAC area above the curve analysis.

2 Value is not different as a proportion of TBW.

In contrast, CD/HFD, HFD/CD, and HFD/HFD F1 males each had increased sums of adipose depots relative to total body weight in F1 male offspring at 21 weeks of age (*P* < 0.05, Fig.1C and D; Table2). The summed adipose depots were greatest in HFD/HFD F1 male offspring in both absolute mass and relative to total body weight compared with all other groups, (*P* < 0.05, Fig.1C and D; Table2). No main effects of F0 diet, F1 diet nor an interaction between the two were evident. F0 and F1 diets did not affect muscle (vastus lateralis or soleus), pancreas or kidney mass in F1 male offspring (*P* > 0.05, Table2). One exception was that in HFD/HFD F1 male offspring livers were heavier compared with CD/CD and HFD/CD F1 (*P* < 0.05, Table2), but were not different as a proportion of total body weight (*P* > 0.05, Table2).

### HFD F0 sired and HFD fed F1 male offspring have the greatest dyslipidemia

F1 male offspring fed a HFD, irrespective of F0 diet (*i.e.,* CD/HFD and HFD/HFD, had elevated fasted serum lipid concentrations (cholesterol, triglyceride, and HDL) compared with CD/CD F1 male offspring (*P* < 0.05, Table2). The greatest dyslipidemia resulted from a paternal HFD combined with an offspring HFD (HFD/HFD) with their serum lipid concentrations exceeding that of CD/HFD and HFD/CD F1 male offspring (*P* < 0.05, Table2). Main effects on F1 male fasted serum cholesterol concentration were evident for F0 HFD on (*P *=* *0.048) and F1 HFD (*P *=* *0.005), but with no interaction between the two (*P *=* *0.282; Table2). The main effect on fasted serum triglycerides was F1 diet (*P *=* *0.040), with no effect of F0 diet (*P *=* *0.059) nor an interaction between the two (*P *=* *0.951; Table2). Main effects on F1 male fasted serum HDL were seen for F1 diet (*P *=* *0.001), but not F0 diet (*P *=* *0.110) nor an interaction between the two (*P *=* *0.330; Table2). Interestingly fasted serum leptin was elevated in F1 males sired by a HFD F0 (*i.e.,* HFD/CD and HFD/HFD), regardless of whether the F1 males they consumed a HFD themselves (Table2). The main effect on fasted serum leptin was F0 diet (*P *=* *0.009), not F1 diet (*P *=* *0.419) nor an interaction between the two (*P *=* *0.289).

### HFD F0 sired and HFD fed F1 male offspring have disturbances in glucose tolerance and insulin sensitivity

Fasting serum insulin and glucose were increased only in HFD/HFD F1 male offspring compared with all other groups (*P *<* *0.05, Table2). A main effect on fasting serum insulin was F0 HFD (*P *=* *0.009), despite no significant effect of F1 diet alone (*P *=* *0.060) and interestingly an interaction between the two (*P = *0.022). However, no main effect was observed for F0 diet (*P = *0.096), F1 diet (*P *=* *0.506) on fasting sera glucose concentrations (interaction *P *=* *0.461).

**Figure 2:**
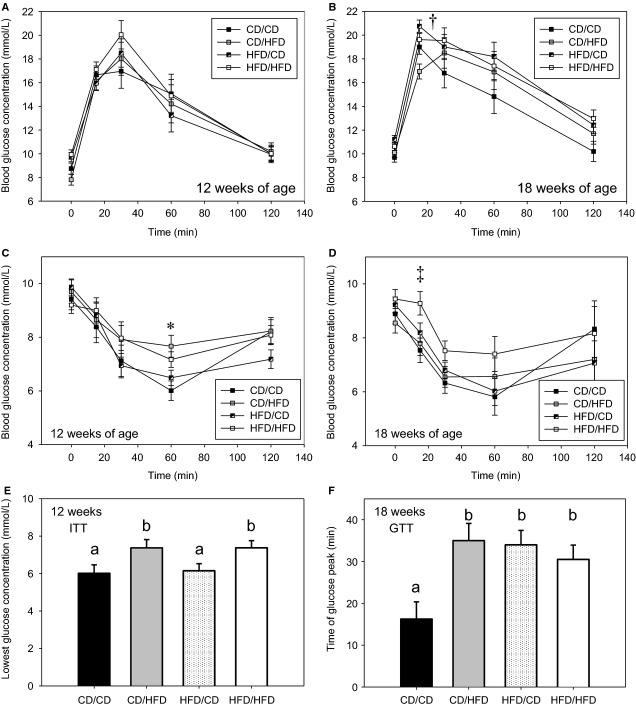
Blood glucose responses to a glucose bolus or insulin challenge administered to F1 male offspring fed a CD/HFD fathered by CD/HFD F0 males. Mean blood glucose responses are depicted for an intraperitoneal glucose tolerance test (GTT) administered at (A) 12 weeks of age; 7 weeks post dietary challenge, (B) 18 weeks of age; 13 weeks post dietary challenge, intraperitoneal insulin tolerance test (ITT) administered at (C) 12 weeks of age; 7 weeks post dietary challenge, (D) 18 weeks of age; 13 weeks post dietary challenge for F1 males, (E) the minimum blood glucose concentration during the ITT at 12 weeks of age and (F) the timepoint at which the peak glucose concentration was achieved during the GTT at 18 weeks of age for the groups CD/CD (CD fed F1 sired by a CD fed F0; *n *=* *7), CD/HFD (HFD fed F1 sired by a CD fed F0; *n *=* *7), HFD/CD (CD fed F1 sired by a HFD fed F0; *n *=* *11) and HFD/HFD (HFD fed F1 sired by a HFD fed F0; *n *=* *11). Data are presented as means ± SEM and different letters/symbols represent statistically distinct groups (*P *<* *0.05). ^†^Delay to peak glucose for CD/HFD, HFD/CD, and HFD/HFD compared to CD/CD (see panel f). *Greater blood glucose concentration at 60 min timepoint for CD/HFD and HFD/HFD compared to CD/CD and HFD/CD (see panel e). ^‡^Increased blood glucose concentration in HFD/HFD compared to all other groups.

No differences were seen between F1 male offspring groups for glucose tolerance (AUC glucose) were evident at either 12 or 18 weeks of age (*P *>* *0.05, Fig.2A and B; Table2). But some evidence of reduced glucose tolerance was observed in CD/HFD, HFD/CD, and HFD/HFD F1 males, evident as a delay to peak blood glucose during the 18 weeks GTT when compared with CD/CD (*P *<* *0.05, Fig.2B and F). Curiously no main effect was found for F0 diet (*P *=* *0.096), F1 diet (*P *=* *0.090), an interaction between both (*P *=* *0.413) nor was it coincident with reduced peak glucose concentration.

**Figure 3:**
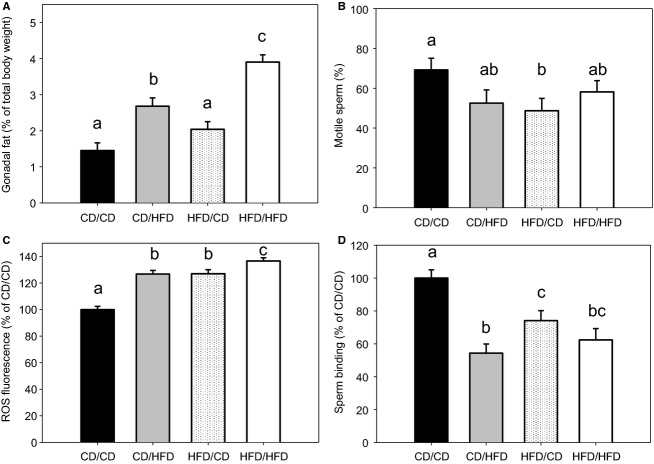
Reproductive measures of F1 male offspring fed a CD/HFD fathered by CD/HFD F0 males. (A) Gonadal fat as a proportion of total body weight, (B) proportion of motile sperm, (C) reactive oxygen species (ROS) as a proportion of CD/CD (*i.e.,* CD/CD set to 100%; using fluorescence readings from an average of *n *=* *41.5 ± 6.6 sperm per group) as determined by DCFDA fluorescence and (D) sperm binding as a proportion of CD/CD (*i.e.,* CD/CD set to 100%; using sperm binding to an average of 34.3 ± 4.0 oocytes per group) are depicted for F1 males for the groups CD/CD (CD fed F1 sired by a CD fed F0; *n *=* *14), CD/HFD (HFD fed F1 sired by a CD fed F0; *n *=* *14), HFD/CD (CD fed F1 sired by a HFD fed F0; *n *=* *20) and HFD/HFD (HFD fed F1 sired by a HFD fed F0; *n *=* *19). Data are presented as means ± SEM and different letters represent statistically distinct groups (*P *<* *0.05).

Insulin sensitivity as measured by AAC was reduced in HFD/HFD F1 males compared with HFD/CD F1 males at both 12 and 18 weeks of age (*P *<* *0.05, Table2) as a response to an insulin bolus. No main effects on AAC was observed for F1 HFD at 12 (*P *=* *0.985) or 18 (*P *=* *0.385) weeks of age, nor due F1 HFD at 12 weeks of age (*P *=* *0.417). But F1 HFD was a main effect by 18 weeks of age (*P *=* *0.045). At both 12 and 18 weeks of age there was no discernible interaction between F0 or F1 diet for AAC (*P *=* *0.403 and *P *=* *0.943, respectively). When assessing insulin tolerance by determining the minimum blood glucose concentration recorded during the 12 weeks of age ITT HFD fed F1 males (CD/HFD and HFD/HFD) had increased blood glucose concentration compared with F1 males fed a CD (CD/CD and HFD/CD, Fig.2E). The main effect on minimum blood glucose concentration during this ITT was F1 diet (*P *=* *0.009) but not F0 diet (*P *=* *0.268) with no interaction between both (*P *=* *0.638). This increased minimum blood glucose concentration is evident at the 60 min timepoint of the 12 weeks of age ITT (Fig.2C, CD/CD: 6.01 ± 0.36 mmol L^−1^, CD/HFD: 7.67 ± 0.41 mmol L^−1^, *P *=* *0.014; HFD/CD: 6.49 ± 0.28 mmol L^−1^, *P *=* *0.323; and HFD/HFD: 7.17 ± 0.29 mmol L^−1^, *P *=* *0.034). But by 18 weeks there was no difference between F1 male groups for the 60 min timepoint. Nor was there any main effects of founder diet (*P *=* *0.570), F1 diet (*P *=* *0.270) nor an interaction between both (*P *=* *0.303) on the minimum sera glucose concentration recorded during the ITT. However, blood glucose concentrations at the 15 min timepoint during the 18 week ITT was elevated in the HFD/HFD F1 males compared with all other groups (Fig.2D, CD/CD: 7.54 ± 0.46 mmol L^−1^; CD/HFD: 7.80 ± 0.46 mmol L^−1^, *P *=* *0.695; HFD/CD: 8.19 ± 0.36 mmol L^−1^, *P *=* *0.292; and HFD/HFD: 9.28 ± 0.44 mmol L^−1^, *P *=* *0.024). This is concordant with their reduced AAC compared to HFD/CD F1 males at this age (Table2). The increased blood glucose at the 15 min timepoint during the 18 week ITT had a main effect of F0 HFD (*P *=* *0.013), F1 HFD (*P *=* *0.044) as an additive effect but not an interaction between them both (*P *=* *0.073).

### A F0 HFD affects the reproductive health of F1 males and is partially compounded by F1 male HFD

Testes weight was not different between F1 male groups (*P *>* *0.05, Table3). HFD/HFD F1 males had heavier seminal vesicles compared with CD/CD males as determined by absolute mass (*P *<* *0.05, Table3), which was normalized when expressed as a proportion of total body weight (Table3). The fat that surrounds the testes (gonadal fat depot) was increased in those offspring fed a HFD irrespective of founder diet (CD/HFD and HFD/HFD) compared with offspring fed a CD (CD/CD and HFD/CD) in terms of both absolute mass and as a proportion of total body weight (*P *<* *0.05, Tables2 and 3). With HFD/HFD F1 offspring also displaying an additive gain in gonadal fat mass compared with CD/HFD F1 (*P < *0.05, Tables2 and 3). There was no main effect of F0 (*P *=* *0.134) or F1 (*P *=* *0.067) diet with regards to gonadal fat mass, nor was an interaction was observed between them (*P *=* *0.466; Tables2 and 3).

**Table 3:** Summary of F_1_ male reproductive measures at age 21 weeks of age, 15 weeks post diet challenge

F0 male diet	CD	CD	HFD	HFD
F1 male diet	CD	HFD	CD	HFD
F_1_ Male offspring	*n *=* *14	*n *=* *14	*n *=* *20	*n *=* *19
Total body weight (g)^1^	36.4 ± 0.8 a	39.8 ± 0.9 bc	37.7 ± 0.8 ab	42.1 ± 0.8 c
Reproductive organs				
Testes (g; L + R)	0.21 ± 0.01	0.20 ± 0.01	0.22 ± 0.01	0.22 ± 0.01
Seminal Vesicles (g; L + R)	0.36 ± 0.03 a	0.38 ± 0.04 ab	0.41 ± 0.03 ab	0.46 ± 0.03 b^2^
Gonadal fat depot (% TBW)	1.45 ± 0.21 a	2.68 ± 0.23 b	2.04 ± 0.21 a	3.91 ± 0.20 c
Sex Hormone				
Testosterone (ng ml^−1^)	2.51 ± 0.53	2.58 ± 0.41	2.41 ± 0.29	3.18 ± 0.28

CD, control diet; HFD, high fat diet.

1 Total body weight (TBW) was measured prior to the overnight fast that preceded post mortem examination. Different letters represent statistically distinct groups (*P *<* *0.05).

2 Value is not significantly different as a proportion of body weight.

Intracellular sperm ROS were elevated in F1 groups either sired by a HFD F0 or fed a HFD (CD/HFD, HFD/CD, and HFD/HFD) compared with CD/CD F1 males (*P* < 0.05, Table3). HFD/HFD F1 males showed a further elevation in sperm ROS compared with CD/HFD and HFD/CD (*P* < 0.05, Table3). This elevation in sperm ROS had a main effect of F0 HFD (*P *<* *0.0001), F1 HFD (*P *<* *0.0001) and an interaction between both (*P *< 0.0001). Sperm–oocyte binding was significantly reduced in CD/HFD, HFD/CD, and HFD/HFD F1 males compared with CD/CD F1 males, and CD/HFD F1 males also reduced compared with HFD/CD but not HFD/HFD (*P* < 0.05, Table3). The main effect on sperm–oocyte binding was F0 diet (*P *=* *0.020), F1 diet (*P *=* *0.020) and an interaction between the two (*P *=* *0.003). Interestingly, the only group who displayed a reduction in sperm motility was the HFD/CD F1 group compared with CD/CD (*P *<* *0.050, Table3). This reduction in sperm motility had a main effect of F0 HFD (*P *=* *0.031), but not F1 diet (*P *=* *0.968) nor an interaction between the two (*P *= 0.086). No differences were detected in the number of sperm extracted from the vas deferens and epididymis nor the proportion of viable sperm between groups (*P *>* *0.05, assessed by PI positive sperm nuclei; data not shown).

## Discussion

Given the current prevalence of overweight and obesity in human populations (Ng et al. 2014) and that eating habits can be transgenerational (Wahlqvist et al. 2014) it is becoming increasingly likely that both parents and children may be exposed to the same obesogenic diet. There is an increasing awareness, at least in the field of maternal programming, that an early life stress exposure increases the risk factors for metabolic disease in later life which can be either manifest or worsened by a secondary stress, a so called “second-hit” phenomenon (Simeoni et al. 2011; Gallo et al. 2012a,b; Li et al. 2013; Cervantes-Rodriguez et al. 2014; Romano et al. 2014). To date this has not been explored in the context of paternal programming. In agreement with other studies, our data demonstrate that both paternal obesity at conception and a consumption of a HFD by an individual animal results in metabolic syndrome and subfertility (Bakos et al. 2011b; Palacios et al. 2011; Fullston et al. 2013; Asrih and Jornayvaz 2014; McPherson et al. 2014). Furthermore, we have determined that the second-hit of the consumption of a HFD by a F1 male that was sired by an obese father exacerbates aspects of these metabolic and sperm derangements. This is the first demonstration of the second hit phenomena in a paternal programming setting.

In the majority of measures presented here the F1 males fed a HFD who were also sired by a HFD F0 had the poorest outcomes. Most striking was the degree of dyslipidemia and increased adiposity observed in HFD fed F1 males sired by obese fathers. These males gained more weight and had significantly more adiposity, showing that there was an additive effect due to their fathers HFD diet combined with own exposure to a HFD. A similar impact was seen for sera lipid concentrations, although curiously only F1 male offspring born to F0s consuming the HFD was hyperleptinemic with no effect of their own consumption of a HFD diet. This finding suggests that these groups potentially have an abnormal adipocyte phenotype (Ogus et al. 2003; Suh et al. 2011) in addition to the increased adiposity observed in these animals. Regardless it remains possible that sera Leptin concentration may have been increased by the continued consumption of HFD by F1 males over a longer period of time. The phenotype of animals born to HFD F0 males is reminiscent of metabolic syndrome that is compounded, in most measures, by F1 male consumption of the same HFD. Indeed HFD/HFD F1 demonstrated some signs of diabetes (*e.g.,* increased sera glucose/insulin concentrations), although insulin secretion tests would be needed to confirm overt diabetes. If translatable to human populations we speculate that a son consuming the same calorie dense diet as his obese father may compound any metabolic pathologies, including increased risk of diabetes, escalating the overall health burden.

The signs of aberrant glucose homeostasis were only unmasked by the second-hit of a HFD combined with their father's consumption of the same HFD. For example, HFD/HFD F1 males demonstrated increased fasted sera glucose concentration and a synergistic increase in sera insulin concentrations, concomitant with insulin resistance (lower AAC for ITT) at both 12/18 weeks of age compared to HFD/CD F1 males. Although it must be noted that the summary measures of insulin sensitivity for HFD/CD and HFD/HFD F1 male groups were not statistically different to the CD/CD and CD/HFD groups. Curiously, these metabolic derangements occurred without any change to the summary measure of glucose tolerance (AUC for GTT) but concomitant with signs of reduced glucose absorbance at 18 weeks of age as evidenced by a delay to peak blood glucose concentrations postglucose bolus.

This second-hit phenomena (Simeoni et al. 2011; Gallo et al. 2012a,b) has been previously described in the setting of maternal nutritional programming (Li et al. 2013; Cervantes-Rodriguez et al. 2014). Using an Agouti mouse model of natural maternal obesity and diabetes with age Li et al. (2013) demonstrated that overt metabolic pathologies were only unmasked in offspring sired by obese/diabetic mothers by their own consumption of a HFD (Li et al. 2013). The second-hit of a HFD in the male offspring born to obese/diabetic mothers led to increased total body weight, hyperleptinemia, glucose intolerance, insulin resistance, increased liver glycerides, and liver steatosis. This phenotype was not observed in either offspring born to obese/diabetic mothers or offspring born to lean mothers fed a HFD (Li et al. 2013). Interestingly Cervantes-Rodriguez et al. (2014) report a similar phenotype in their offspring (increased triglycerides, insulin, cholesterol, leptin, and adiposity) due to a second-hit of sugar consumption following the first hit of maternal protein restriction. In this case the offspring phenotype due to maternal protein restriction was exacerbated by the consumption of 5% sucrose in drinking water beyond the individual effects seen for either being born to a protein restricted mother or the consumption of the sugar water alone (Cervantes-Rodriguez et al. 2014). Here, we report a similar phenomenon, with the male offspring phenotype worsened by the second-hit of the consumption of a HFD with the “first-hit” resulting from preconception paternal obesity/HFD.

It has been previously demonstrated that the reproductive function of F1 males is impaired by diet-induced obesity of the father (Fullston et al. 2012; McPherson et al. 2014). Here, we recapitulate these findings demonstrating reductions in sperm motility, sperm binding, and increases in oxidative stress. Furthermore, similar to other studies, feeding a HFD to a male also impaired sperm binding and increased oxidative stress (Bakos et al. 2011b; Palmer et al. 2011; Fullston et al. 2012). Of particular interest was the significant interaction between an obese father and male offspring consuming a HFD as an adolescent/adult on sperm function. The sperm of these F1 males had aberrant measures of sperm binding and oxidative stress. Elevated oxidative stress in sperm is associated with subfertility (Bakos et al. 2011a; Tunc et al. 2011; Fullston et al. 2012; Sermondade et al. 2012) and increases in oxidative DNA lesions and DNA damage in sperm (Weitzman et al. 1994; Aitken et al. 2014), which are further implicated as markers of reduced pregnancy and increased miscarriage rates (Lewis and Aitken 2005; Bakos et al. 2008). Furthermore, reductions in sperm binding suggest that there may be amplification in sperm morphological anomalies with consumption of a HFD diet and an obese father. During epididymal transit sperm gain motility and fertilizing capability with the incorporation of significant amounts of proteins and microRNA, in addition to vast remodeling of the sperm surface/membrane composition (Frenette et al. 2002; Dacheux and Dacheux 2014). Thus, the epipididymal environment within these HFD/HFD F1 males may have been further perturbed from the consumption of a HFD resulting in a further reduction in sperm function.

Although the most severe sperm impairment was evident in HFD/HFD F1 males, the exacerbation of paternal HFD-induced phenotypes by offspring HFD feeding is not consistent across the measures examined here. For example gonadal adiposity was significantly greater in the HFD/HFD F1 males in a manner that was additive of father's HFD and males offspring's HFD. Whereas sperm ROS concentrations and sperm–oocyte binding both had significant effects due to a father's or an offpsring's HFD alone and an interaction between them. It must be noted that a broad range of ROS was measured here and that to identify which specific subspecies might be responsible would require further investigation. Regardless, elevated sperm ROS concentrations can induce metabolic derangements in offspring (Lane et al. 2014a) possibly resulting from increased oxidative damage to DNA (Weitzman et al. 1994; Noblanc et al. 2013) which may be more prone to mismatch repair and increasing the mutation load in embryo and offspring (Aitken et al. 2014). This perhaps suggests that the health of the F2 generation is at greater risk of health pathologies, due to the combined effects of F0 and F1 male HFD, and potentially resulting in an acceleration to the transgenerational amplification of obesity, metabolic disease, and subfertility.

In contrast sperm motility and sera testosterone concentration was not further impaired by the combination of both a father and an offspring consuming the same HFD. In fact F1 male HFD feeding, regardless of F0 diet, resulted in sperm motility that was not different to control F1 animals. These effects did not align with gonadal adiposity and implies that increased scrotal heat, that might result from increased gonadal adiposity (Shafik and Olfat 1981; Setchell 1998), does not impair these measures further, but may still relate to sperm ROS. Although not significantly improved, perhaps the effect of HFD-induced paternal programming has a somewhat protective effect in F1 male offspring when exposed to the same HFD in regards to sperm motility. Indeed the worst sperm motility was observed for HFD/CD F1 males, potentially implying that this combination dietary mismatch between father/son had a greater impact than F1 males with either matching father/son diets or CD/HFD F1 males. Furthermore, the consumption of a HFD by F1 males born to obese fathers had less impact on both oxidative stress and sperm binding compared to those born to lean fathers. Whether these sperm measures in HFD born F1 males are an artifact of a threshold effect or potential evidence of a predictive adaptive response warrants further investigation.

The precise mechanism(s) underlying paternal programming remains unknown, but inherited aberrant epigenetic profiles transmitted by sperm are clearly implicated as potential mediators (Youngson and Whitelaw 2011; Lane et al. 2014b; Ost et al. 2014). Indeed paternal HFD has been demonstrated to alter sperm microRNA content (Fullston et al. 2013), global methylation in mature sperm cells (Fullston et al. 2013) and acetylation of histones prior to protamination (Palmer et al. 2011). Whether aberrant sperm epigenetic profiles harbored by the sperm of HFD founder males are faithfully replicated in offspring or lead to dysregulation of key developmental processes that result in a different, but still perturbed, epigenetic profile in offspring remains to be determined. Moreover, whether any aberrant epigenetic profiles in F1 male offspring are further disrupted by their HFD feeding remains to be investigated.

We demonstrate for the first time that the double hit of an F1 male being exposed to the same obesogenic diet as his father exacerbates the effects of HFD-induced paternal programming of metabolic derangements and diminished reproductive measures. The magnitude of the second-hit was not consistent between measures that were affected, ranging from no effect to synergistic of the individual effects seen for each a F0's HFD or a F1 male's HFD. Furthermore, the second hit revealed signs of dysregulated glucose homeostasis, not evident in either of the single hit groups and the greatest reproductive impairment. Overall, we provide evidence for the potential of a common obesogenic diet shared between a father and his son to accelerate the transgenerational amplification of obesity, metabolic syndrome, and diminished reproductive capacity. If translatable to human health setting, this provides a sombre warning in the midst of the current global human obesity epidemic.
